# Two new species of Ophiostomatales (Sordariomycetes) associated with the bark beetle *Dryocoetes alni* from Poland

**DOI:** 10.3897/mycokeys.68.50035

**Published:** 2020-06-17

**Authors:** Beata Strzałka, Robert Jankowiak, Piotr Bilański, Nikita Patel, Georg Hausner, Riikka Linnakoski, Halvor Solheim

**Affiliations:** 1 Department of Forest Ecosystems Protection; University of Agriculture in Krakow, Al. 29 Listopada 46, 31-425, Krakow, Poland University of Agriculture Kraków Poland; 2 Department of Microbiology, Buller Building 213, University of Manitoba, Winnipeg, R3T 2N2, Canada University of Manitoba Winnipeg Canada; 3 Natural Resources Institute Finland (Luke), Latokartanonkaari 9, 00790, Helsinki, Finland Natural Resources Institute Finland Helsinki Finland; 4 Norwegian Institute of Bioeconomy Research, P.O. Box 115, 1431, Ås, Norway Norwegian Institute of Bioeconomy Research Ås Norway

**Keywords:** Bark beetle, *
Ceratocystiopsis
*, hardwoods, *
Leptographium
*, ophiostomatoid fungi, taxonomy, two new species

## Abstract

Bark beetles belonging to the genus *Dryocoetes* (Coleoptera, Curculionidae, Scolytinae) are known vectors of fungi, such as the pathogenic species *Grosmannia dryocoetidis* involved in alpine fir (*Abies lasiocarpa*) mortality. Associations between hardwood-infesting *Dryocoetes* species and fungi in Europe have received very little research attention. Ectosymbiotic fungi residing in *Ceratocystiopsis* and *Leptographium* (Ophiostomatales, Sordariomycetes, Ascomycota) were commonly detected in previous surveys of the *Dryocoetes alni*-associated mycobiome in Poland. The aim of this study was to accurately identify these isolates and to provide descriptions of the new species. The identification was conducted based on morphology and DNA sequence data for six loci (ITS1-5.8S, ITS2-28S, ACT, CAL, TUB2, and TEF1-α). This revealed two new species, described here as *Ceratocystiopsis synnemata***sp. nov.** and *Leptographium alneum***sp. nov**. The host trees for the new species included *Alnus incana* and *Populus tremula*. *Ceratocystiopsis synnemata* can be distinguished from its closely related species, *C. pallidobrunnea*, based on conidia morphology and conidiophores that aggregate in loosely arranged synnemata. *Leptographium alneum* is closely related to *Grosmannia crassivaginata* and differs from this species in having a larger ascomatal neck, and the presence of larger club-shaped cells.

## Introduction

Bark beetles in the genus *Dryocoetes* (Coleoptera: Curculionidae: Scolytinae) are mainly secondary pests infesting dead, injured, and felled or windthrown conifer- and hardwood hosts. For this reason, most members of *Dryocoetes* have no or only minor economic importance, although *Dryocoetes confusus*, the most destructive species in the genus, may cause extensive mortality of subalpine fir (*Abies lasiocarpa*) in North America (Bright 1963; Negrón and Popp 2009). The biology of hardwood-infesting *Dryocoetes* species is poorly understood in Poland. One of them is *Dryocoetes alni* (Georg), which has a wide geographical distribution, extending from France in the west to Siberia in the east, and from Fennoscandia in the north to Italy and Asia Minor in the south (Dodelin 2010). In Poland it occurs rarely, but probably it is widespread. This beetle species attacks weakened or dead trees of *Alnus* spp., *Populus* spp. and *Corylus avellana* (Gutowski and Jaroszewicz 2001; Borowski et al. 2012).

*Dryocoetes* beetles live in close association with fungi; most notably with members of the Ophiostomatales (Ascomycota, Sordariomycetes) that are well-recognized associates of bark- and wood-dwelling beetles (Kirisits 2004; Wingfield et al. 2017). According to Six (2012), associations among bark beetles and fungi range from mutualistic to commensal, and from facultative to obligate. Some fungi are highly specific and associated only with a single beetle species, while others can be associated with many beetle species. The majority of fungi vectored by *Dryocoetes* cause sapstain but some are responsible for serious tree diseases, such as the pathogenic species of *Leptographium*, *Grosmannia dryocoetidis* which is involved in *A. lasiocarpa* mortality (Molnar 1965). Ophiostomatales is comprised of two families, Kathistaceae and Ophiostomataceae, the latter comprising several several phylogenetic lineages that include, among others, *Ceratocystiopsis*, *Graphilbum*, *Leptographium*, *Ophiostoma*, *Raffaelea* and *Sporothrix* (Hyde et al. 2020). Members of these lineages have similar morphological and ecological characteristics. These fungi are also referred to as so-called ophiostomatoid fungi, a polyphyletic grouping characterized by the production of sticky spore masses at the apices of the flask-shaped sexual fruiting structures and their association with different arthropods (De Beer and Wingfield 2013; De Beer et al. 2013a, b, 2016; Hyde et al. 2020).

*Leptographiumsensu lato* is a broadly defined polyphyletic group of morphologically similar species (De Beer and Wingfield 2013). To date, *Leptographiumsensu lato* includes ten species complexes and some smaller lineages with uncertain taxonomic positions (De Beer and Wingfield 2013; Jankowiak et al. 2017). The genus *Leptographium* contains more than 150 described taxa, most of which are associated with phloem- and wood breeding beetles that affect a wide range of plants worldwide (Jacobs and Wingfield 2001). *Leptographium* species colonizing the roots of conifers may cause tree health problems, such as members of the *Leptographium wageneri* species complex that are responsible for black stain root disease (BSRD) on conifers in western North America (Goheen and Cobb 1978). Morphologically, species of *Leptographiumsensu lato* are characterized by mononematous, darkly pigmented conidiophores terminating in penicillate branches. In addition, species belonging to the *Grosmannia olivacea* species complex also form synnematous conidiophores. Some members of *Leptographiumsensu lato* produce sporothrix-like or hyalorhinocladiella-like synanamorphs. Many *Leptographiumsensu lato* also form sexual morphs characterized by globose ascomata with elongated necks (Jacobs and Wingfield 2001) and these were often included in the genus *Grosmannia* Goid. (Goidànich 1936).

*Leptographium* species have historically been classified into various genera including *Grosmannia*, *Ceratocystis* Ellis and Halst. (Upadhyay 1981), and *Ophiostoma* Syd. and P. Syd. (Seifert et al. 1993). Phylogenetic analyses based on the ribosomal large subunit (LSU) and beta-tubulin sequence data carried out by Zipfel et al. (2006) documented distinct differences between *Ophiostoma* and *Grosmannia*, and redefined the latter genus to include all *Leptographium* with sexual morphs. De Beer and Wingfield (2013) re-evaluated the taxonomy of *Leptographium* and *Grosmannia*, considering all available DNA sequence data for all species. The authors concluded that sequence data for additional gene regions would be necessary to fully resolve the delineation of *Leptographium* and *Grosmannia*. De Beer and Wingfield (2013) suggested that all known *Leptographium* and *Grosmannia* placed in *Leptographiumsensu lato* based on phylogenetic inference, should be treated in their current genera (*Leptographium* or *Grosmannia*). However, new species, excluding those residing in the *G. penicillata* species complex, should provisionally be treated in *Leptographium*, irrespective of their sexual or asexual morphs.

In contrast to species of *Leptographiumsensu lato*, members of *Ceratocystiopsis* are less widespread globally. The genus *Ceratocystiopsis* currently includes nearly 20 taxa, most of which are collected from plants infested by phloem and wood-breeding beetles. *Ceratocystiopsis* species have short-necked perithecia, elongated ascospores, and hyalorhinocladiella-like asexual morphs (Upadhyay 1981; De Beer and Wingfield 2013).

Surveys of hardwood-infesting bark beetles in Poland have recently led to the recovery of an unknown *Leptographium* species from *Dryocoetes alni* (Jankowiak et al. 2019a). In addition, several isolates resembling *Ceratocystiopsis* have also been isolated from *D. alni* in association with *Populus tremula* L. In this study, all known *Leptographium* and *Ceratocystiopsis* species as well as the newly collected isolates were compared based on morphology and DNA sequence data for six nuclear loci, with the overall aim of providing accurate identifications for these fungi.

## Materials and methods

### Isolates and herbarium specimens

Isolations were made from the bark beetle *D. alni* and its galleries established in *P. tremula* logs. Strains were collected in beech-alder stand in southern Poland (Paprocice: 50°48'56.10"N, 21°2'51.23"E) during March–September 2018. The isolation procedures were the same as described by Jankowiak et al. (2019a). Isolates were also collected from *Alnus incana* (L.) Moench and *P. tremula* infested by *D. alni* and from *Malus sylvestris* (L.) Mill. infested by *Scolytus mali* (Bechstein) during studies conducted by Jankowiak et al. (2019a).

All fungal isolates used in this study are listed in Table 1. The isolates are maintained in the culture collection of the Department of Forest Pathology, Mycology and Tree Physiology; University of Agriculture in Krakow, Poland, and in the culture collection of the Natural Resources Institute Finland (Luke), Finland. The ex-type isolates of the new species described in this study were deposited in the Westerdijk Fungal Biodiversity Institute (**CBS**), Utrecht, the Netherlands, and in the culture collection (**CMW**) of the Forestry and Agricultural Biotechnology Institute (**FABI**), University of Pretoria, South Africa. Herbarium specimens have been deposited in the Herbarium of the University of Turku (**TUR**), Finland. Three reference strains were obtained from collections. These included a living culture of *Ceratocystiopsis pallidobrunnea* (WIN(M)51) from the culture collection of University of Manitoba (Canada), and cultures of *Grosmannia crassivaginata* (CBS 119444) and *Leptographium piriforme* (CMW 52066) (Table 1). Taxonomic descriptions and nomenclatural data have been registered in MycoBank (www.MycoBank.org) (Robert et al. 2013).

**Table 1.** Fungal isolates used in the present study.

Species^1^	Isolate no^2^			Herbarium no^3^	Host	Insect vector	Origin	GenBank accession no^4^					
	CMW	CBS	KFL and NRIF	TUR				ITS1-5.8S-ITS2-28S	ITS2-28S	TUB2	TEF1-α	ACT	CAL
Taxon 1													
***Ceratocystiopsis synnemata* sp. nov.**			16216DA	http://mus.utu.fi/TFU.207991	*Alnus incana*	*Dryocoetes alni*	Resko	**MN900984**		**MN901005**	**MN901014**	not obtained	not obtained
			13418DA	http://mus.utu.fi/TFU.207992	*Populus tremula*	*Dryocoetes alni*	Paprocice	**MN900985**		**MN901006**	**MN901015**	not obtained	not obtained
			149a18DA	http://mus.utu.fi/TFU.207993	*Populus tremula*	*Dryocoetes alni*	Paprocice	**MN900986**		**MN901007**	**MN901016**	not obtained	not obtained
			149b18DA	http://mus.utu.fi/TFU.207994	*Populus tremula*	*Dryocoetes alni*	Paprocice	**MN900987**		**MN901008**	**MN901017**	not obtained	not obtained
			16918DA^H^	http://mus.utu.fi/TFU.207995	*Populus tremula*	*Dryocoetes alni*	Paprocice	**MN900988**		**MN901009**	**MN901018**	not obtained	not obtained
			17718DA	http://mus.utu.fi/TFU.207996	*Populus tremula*	*Dryocoetes alni*	Paprocice	**MN900989**		**MN901010**	**MN901019**	not obtained	not obtained
Taxon 2													
***Leptographium alneum* sp. nov.**	52067	144905	13116RJDA	http://mus.utu.fi/TFU.207559	*Alnus incana*	*Dryocoetes alni*	Resko	**MN900990**	MH283185	MH283218	MH283406	**MN901029**	**MN901041**
	52072	144904	16016bRJDA	http://mus.utu.fi/TFU.207997	*Alnus incana*	*Dryocoetes alni*	Resko	**MN900991**		MH283219	MH283407	**MN901030**	**MN901042**
		144903	16216bRJDA	http://mus.utu.fi/TFU.207998	*Alnus incana*	*Dryocoetes alni*	Resko	**MN900992**	MH283186	MH283220	MH283408	**MN901031**	**MN901043**
	52070		7617RJDA		*Populus tremula*	*Dryocoetes alni*	Paprocice	**MN900993**		MH283221	**MN901020**	**MN901032**	**MN901044**
	52075	144902	7717RJDA	http://mus.utu.fi/TFU.207558	*Populus tremula*	*Dryocoetes alni*	Paprocice	**MN900994**		MH283222	**MN901021**	**MN901033**	**MN901045**
	52069		8417RJDA		*Populus tremula*	*Dryocoetes alni*/	Paprocice	**MN900995**		MH283223	**MN901022**	**MN901034**	**MN901046**
			8617RJDA		*Populus tremula*	*Dryocoetes alni*	Paprocice	**MN900996**		MH283224	**MN901023**	**MN901035**	**MN901047**
	52076^H^	144901^H^	8917RJDA^H^	http://mus.utu.fi/TFU.207557	*Populus tremula*	*Dryocoetes alni*	Paprocice	**MN900997**		MH283225	**MN901024**	**MN901036**	**MN901048**
			9117RJDA		*Populus tremula*	*Dryocoetes alni*	Paprocice	**MN900998**		MH283226	**MN901025**	**MN901037**	**MN901049**
			88616RJSM		*Malus sylvestris*.	*Scolytus mali*	Rozpucie	**MN900999**	MH283187	MH283227	MH283409	**MN901038**	**MN901050**
	52071	144900	88716aRJSM	http://mus.utu.fi/TFU.207556	*Malus sylvestris*	*Scolytus mali*	Rozpucie	**MN901000**	MH283188	MH283228	MH283410	**MN901039**	**MN901051**
*Leptographium piriforme*	52066		297NBRZ16AO		*Betula pendula*	Wound	Żohatyn	**MN901001**	MH740931	MH740984	MH741134	not obtained	not obtained
			10618DA		*Alnus incana*	*Dryocoetes alni*	Paprocice	**MN901002**		**MN901011**	**MN901026**	not obtained	not obtained
*Grosmannia crassivaginata*	134	119144			unknown	unknown	unknown	**MN901003**		**MN901012**	**MN901027**	**MN901040**	not obtained
*Ceratocystiopsis pallidobrunnea*			WIN(M) 51		*Populus tremuloides*	unknown	Duck Mountain^5^	**MN901004**		**MN901013**	**MN901028**	not obtained	not obtained

^1^Boldtype = new species in the present study. ^2^CMW Culture Collection of the Forestry and Agricultural Biotechnology Institute (FABI), University of Pretoria, Pretoria, South Africa; CBS Westerdijk Fungal Biodiversity Institute, Utrecht, The Netherlands; KFL Culture collection of the Department of Forest Pathology, Mycology and Tree Physiology; University of Agriculture in Krakow, Poland; NRIF The Natural Resources Institute Finland (Luke), Helsinki, Finland; WIN the University of Manitoba (Winnipeg); Canada. H = ex-holotype ^3^TFU the TUR Herbarium of the University of Turku, Finland ^4^ITS1-5.8S-ITS2-ITS2-28S = the internal transcribed spacer 1 and 2 regions of the nuclear ribosomal DNA gene, 5.8S rRNA gene; and the 28S large subunit of the nrDNA gene; ACT= Actin; TUB2 = Beta-tubulin; CAL= Calmodulin; TEF1-α = Translation elongation factor 1-alpha; **Bold** type = GenBank accession numbers of sequences obtained in the present study. ^5^Duck Mountain Provincial Forest, Manitoba, Canada.

### DNA extraction, PCR and sequencing

DNA extractions were done as described by Jankowiak et al. (2018). For sequencing and phylogenetic analyses, six loci were amplified: internal transcribed spacer 1 and 2 (ITS1-5.8S-ITS2), internal transcribed spacer 2 and large subunit (ITS2-28S), actin (ACT), beta-tubulin (TUB2), calmodulin (CAL) and the translation elongation factor 1-alpha (TEF1-α) using the primers listed in Table 2.

**Table 2.** Information on PCR primers used in this study.

Locus	Primers	Fungi
ITS1-5.8S	ITS1-F (Gardes and Bruns 1993), ITS4 (White et al. 1990)	*Ceratocystiopsis*, *Leptographium*
28S	LR0R, LR5 (Vilgalys and Hester 1990)	* Ceratocystiopsis *
ITS2-28S	ITS3 (White et al. 1990), LR3 (Vilgalys and Hester 1990)	* Leptographium *
TUB2	Bt2a, Bt2b (Glass and Donaldson 1995)	* Ceratocystiopsis *
	T10 (O’Donnell and Cigelnik 1997), Bt2b (Glass and Donaldson 1995)	* Leptographium *
ACT	Lepact-F, Lepact-R (Lim et al. 2004)	* Leptographium *
CAL	CL3F, CL3R (De Beer et al. 2016)	* Leptographium *
TEF1-α	F-728F (Carbone and Kohn 1999), EF2 (O’Donnell et al. 1998)	* Ceratocystiopsis *
	EF1F, EF2R (Jacobs et al. 2004)	* Leptographium *

DNA fragments were amplified in a 25 µL reaction mixture containing 0.25 µL of Phusion High-Fidelity DNA polymerase (Finnzymes, Espoo, Finland), 5 µL Phusion HF buffer (5x), 0.5 µL of dNTPs (10 mM), 0.75 µL DMSO (100%) and 0.5 µL of each primer (25 µM). Amplification reactions were performed in the LabCycler Gradient thermocycler (Sensoquest Biomedical Electronics GmbH, Germany). Amplification of the various loci was performed under the following conditions: a denaturation step at 98 °C for 30 s was followed by 35 cycles of 5 s at 98 °C, 10 s at 52–64 °C (depending on the primer melting temperature and fungal species) and 30 s at 72 °C, and a final elongation step at 72 °C for 8 min. The PCR products were visualized under UV light on a 2% agarose gel stained with Midori Green DNA Stain (Nippon Genetic Europe).

Amplified products were sequenced with the BigDye Terminator v 3.1 Cycle Sequencing Kit (Applied Biosystems, Foster City, CA, USA) and the products were resolved with an ABI PRISM 3100 Genetic Analyzer (Applied Biosystems), at the DNA Research Centre (Poznań, Poland) using the same primers that were used for the PCR. The sequences (Table 1) were compared with sequences retrieved from GenBank using the BLASTn algorithm (Altschul et al. 1990). Newly obtained sequences were deposited in NCBI GenBank and added to previous alignments for the ITS1-5.8S-ITS2-28S and ITS2-28S regions (Jankowiak et al. 2019a). Alignments were adjusted to accommodate the new sequences and the data sets were used to obtain consensus sequences and the two data sets were concatenated.

### Phylogenetic analyses

**Figure 1.**
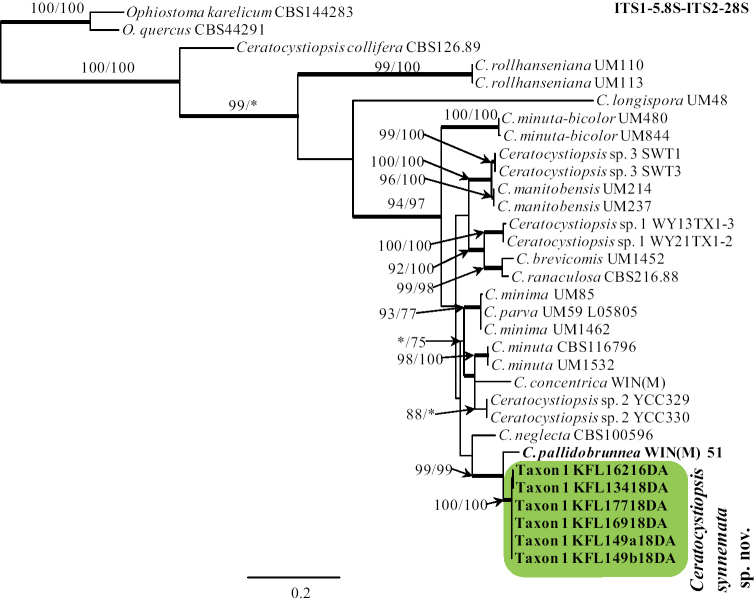
Phylogram obtained from Maximum Likelihood (ML) analyses of the ITS1-5.8S-ITS2-28S data for the *Ceratocystiopsis* spp. Sequences obtained during this study are presented in bold type. The Bootstrap values ≥ 75% for ML and Maximum Parsimony (MP) analyses are presented at nodes as follows: ML/MP. Bold branches indicate posterior probabilities values ≥ 0.95 obtained from Bayesian Inference (BI) analyses. * Bootstrap values <75%. The tree is drawn to scale (see bar) with branch length measured in the number of substitutions per site. *Ophiostoma karelicum* and *Ophiostoma quercus* represent the outgroup.

**Figure 2.**
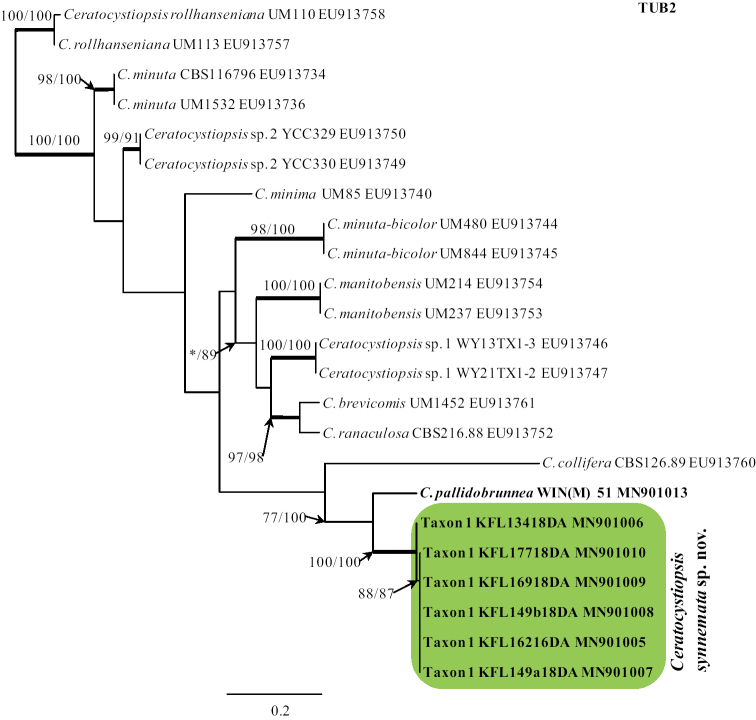
Phylogram obtained from Maximum Likelihood (ML) analyses of TUB2 data for the *Ceratocystiopsis* spp. Sequences obtained during this study are presented in bold type. The Bootstrap values ≥ 75% for ML and Maximum Parsimony (MP) analyses are presented at nodes as follows: ML/MP. Bold branches indicate posterior probabilities values ≥ 0.95 obtained from Bayesian Inference (BI) analyses. * Bootstrap values <75%. The tree is drawn to scale (see bar) with branch length measured in the number of substitutions per site.

**Figure 3.**
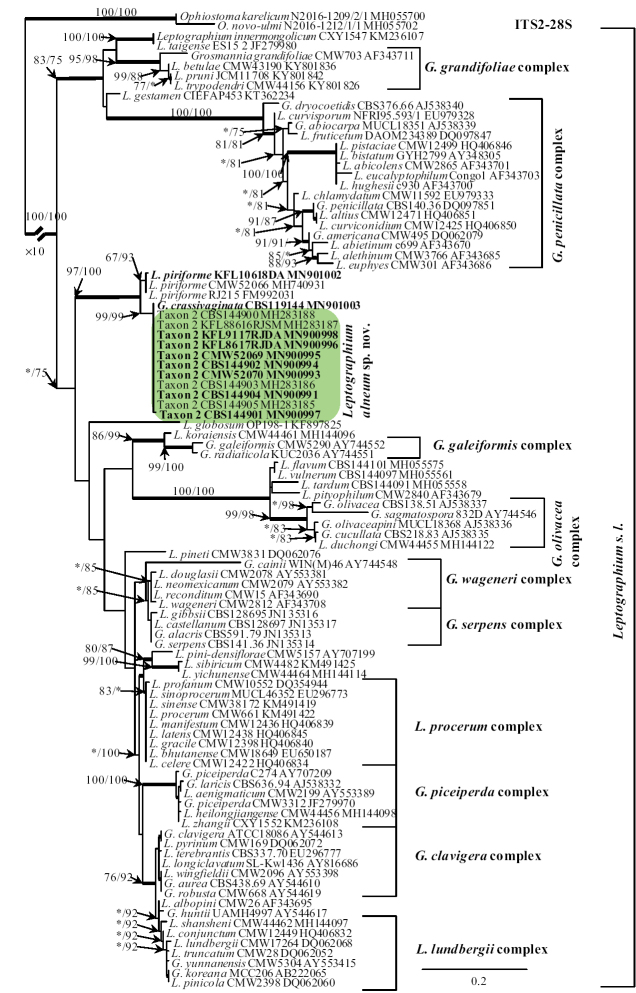
Phylogram obtained from Maximum Likelihood (ML) analyses of the ITS2-28S for selected species of *Leptographiumsensu lato*. Sequences obtained during this study are presented in bold type. The Bootstrap values ≥ 75% for ML and Maximum Parsimony (MP) analyses are presented at nodes as follows: ML/MP. Bold branches indicate posterior probabilities values ≥ 0.95 obtained from Bayesian Inference (BI) analyses. * Bootstrap values <75%. The tree is drawn to scale (see bar) with branch length measured in the number of substitutions per site. *Ophiostoma karelicum* and *O. quercus* represents the outgroup in analyses of ITS2-28S.

**Figure 4.**
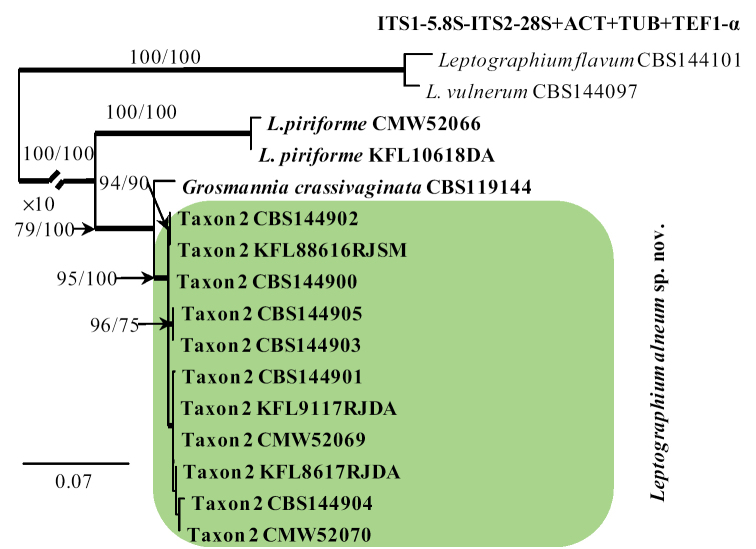
Phylogram obtained from Maximum Likelihood (ML) analyses of the combined datasets of ITS1-5.8S-ITS2-28S+ACT+TUB2+TEF1-α for selected species of *Leptographiumsensu lato*. Sequences obtained during this study are presented in bold type. The Bootstrap values ≥ 75% for ML and Maximum Parsimony (MP) analyses are presented at nodes as follows: ML/MP. Bold branches indicate posterior probabilities values ≥ 0.95 obtained from Bayesian Inference (BI) analyses. * Bootstrap values <75%. The tree is drawn to scale (see bar) with branch length measured in the number of substitutions per site. *Leptographium flavum* and *L. vulnerum* represents the outgroup in analyses of the combined datasets of ITS1-5.8S-ITS2-28S+ACT+TUB2+TEF1-α.

BLAST searches (Altschul et al. 1990) using the BLASTn algorithm were performed to retrieve similar sequences from GenBank (http://www.ncbi.nlm.nih.gov) and accession numbers for these sequences are presented in the corresponding phylogenetic trees (Figs 1–4). Datasets were curated with the Molecular Evolutionary Genetic Analysis (MEGA) v6.06 program (Tamura et al. 2013).

The phylogenetic position of Taxon 1 was determined from their concatenated ITS1-5.8S-ITS2-28S sequences within a dataset that covered all ITS1-5.8S and ITS2-28S sequences of *Ceratocystiopsis* available in GenBank, as well as sequences of *C. pallidobrunnea* obtained in this study (Fig. 1). The outgroup taxa for the ITS1-5.8S-ITS2-28S dataset analysis were *Ophiostoma karelicum* and *O. quercus*. The TUB2 dataset included all available sequences for reference species in *Ceratocystiopsis* that could be retrieved from GenBank and six of our isolates (Fig. 2) in order to identify isolates to the species level.

In the case of Taxon 2, the ITS2–28S dataset included most of the available sequences for reference species in *Leptographiumsensu lato* that could be retrieved from GenBank (Fig. 3) to show the placement of our isolates within this group. The outgroup taxa for the ITS2–28S dataset analysis was *O. karelicum* and *O. novo-ulmi*. The concatenated constructs of sequences for multiple loci (ITS1-5.8S-ITS2–28S + TUB2 + TEF1-α + ACT) were also used for 11 of our isolates, *Grosmannia crassivaginata*, and *L. piriforme* (Fig. 4). Before individual data sets for the ITS2-28S, ACT, TUB2, and the TEF1-α gene regions were used for 11 of our isolates, *Grosmannia crassivaginata*, and *L. piriforme*. The outgroup taxa for the ITS1-5.8S-ITS2–28S + TUB2 + TEF1-α + ACT datasets analysis were *Leptographium flavum* and *L. vulnerum*. Datasets concerning the protein coding sequences were concatenated. Sequence alignments were performed using the online version of MAFFT v7 (Katoh and Standley 2013). The ITS1-5.8S, ITS2-28S, ACT, TUB2, and TEF1-α datasets were aligned using the E-INS-i strategy with a 200PAM/k=2 scoring matrix, a gap opening penalty of 1.53 and an offset value of 0.00. The alignments were checked manually with BioEdit v.2.7.5 (Hall 1999). The resulting alignments and trees were deposited into TreeBASE (http://purl.org/phylo/treebase/phylows/study/TB2:S25615). Aligned data sets of the protein-coding genes were compared to gene maps constructed by Yin et al. (2015) to determine the presence or absence of introns and to confirm that introns and exons were appropriately aligned (Suppl. material 1: Tables S1–S3). Single nucleotide polymorphisms (SNPs) for different gene regions between the new taxa and the phylogenetically closest related species were also identified by comparative sequence analysis.

Phylogenetic trees were inferred for each of the datasets using three different methods: Maximum likelihood (ML), Maximum Parsimony (MP) and Bayesian inference (BI). For ML and BI analyses, the best-fit substitution models for each aligned dataset were established using the corrected Akaike Information Criterion (AICc) in jModelTest 2.1.10 (Guindon and Gascuel 2003; Darriba et al. 2012). ML analyses were carried out with PhyML 3.0 (Guindon et al. 2010), utilizing the Montpelier online server (http://www.atgc-montpellier.fr/phyml/). The ML analysis included bootstrap analysis (1000 bootstrap pseudoreplicates) in order to assess node support values and the overall reliability of the tree topology.

The best evolutionary substitution model for ITS2-28S (*Leptographium*) and the ITS1-5.8S-ITS2–28S (*Ceratocystiopsis*) was GTR+I+G. The best evolutionary substitution model for TUB2 (*Ceratocystiopsis*) and for the combined ITS1-5.8S-ITS2–28S, ACT, TUB2, and TEF1-α, datasets for *Leptographium* was GTR+G.

MP analyses were performed with PAUP* 4.0b10 (Swofford 2003). Gaps were treated as fifth state. Bootstrap analysis (1000 bootstrap replicates) was conducted to determine the levels of confidence for the nodes within the inferred tree topologies. Tree bisection and reconnection (TBR) was selected as the branch swapping option. The tree length (TL), Consistency Index (CI), Retention Index (RI), Homoplasy Index (HI) and Rescaled Consistency Index (RC) were recorded for each analysed dataset after the trees were generated.

BI analyses using Markov Chain Monte Carlo (MCMC) methods were carried out with MrBayes v3.1.2 (Ronquist and Huelsenbeck 2003). The four MCMC chains were run for 10 million generations applying the best-fit model for each data set. Trees were sampled every 100 generations, resulting in 100,000 trees. The Tracer v1.4.1 program (Rambaut and Drummond 2007) was utilized to determine the burn-in value for each dataset. The remaining trees were utilized to generate a 50% majority rule consensus tree, which allowed for calculating posterior probability values for the nodes.

### Morphology, growth studies and mating tests

Morphological characters were examined for selected isolates and for the herbarium specimens chosen to represent the type specimens for the newly proposed species. Cultures were grown on 2% MEA agar [MEA: 20 g Bacto malt extract (Becton Dickinson and Company, Franklin Lakes, USA), 20 g agar (Bacto agar powder from Becton Dickinson and Company, Franklin Lakes, USA), 1 l deionized water] with or without host tree twigs to induce potential ascocarp formation. Autoclaved twigs with bark were positioned in the centre of the MEA agar plates. Fungal cultures were derived from single spores, and crossings were made following the technique described by Grobbelaar et al. (2010). To encourage the production of ascomata for species descriptions, single conidial isolates were crossed in all possible combinations. Cultures were incubated at 25 °C and monitored regularly for the appearance of fruiting structures.

Morphological features were examined by mounting materials in 80% lactic acid on glass slides, and observing various fruiting structures using a Nikon Eclipse 50*i* microscope (Nikon Corporation, Tokyo, Japan) with an Invenio 5S digital camera (DeltaPix, Maalov, Denmark) to capture photographic images. Microscopy was done as previously described by Kamgan Nkuekam et al. (2011). Color designations were based on the charts of Kornerup and Wanscher (1978).

For each taxonomically relevant structure fifty measurements were made, whenever possible, with the Coolview 1.6.0 software (Precoptic, Warsaw, Poland). Averages, ranges and standard deviations were calculated for the measurements, and these are presented in the format ‘(min–)(mean-SD)–(mean+SD)(–max)’.

Growth characteristics for the two newly proposed species and *Grosmannia crassivaginata* (isolate CBS 119144) were determined by analyzing the radial growth for five isolates in pure culture that represent each of the studied species (Table 1). Agar disks (5 mm diam.) were cut from actively growing margins of fungal colonies for each of the tested isolates and these disks were placed in the center of plates containing 2% MEA. Four replicate plates for each of the proposed new species and *G. crassivaginata* were incubated at 5, 10, 15, 20, 25, 30 and 35 °C. The radial growth (two measurements per plate) were determined 7 d (Taxon 1) and 4 d (Taxon 2, and *G. crassivaginata*) after inoculation, and growth rates were calculated as mm/d.

## Results

### Morphological characteristics

The two new taxa showed differences with regards to growth rates in culture and color differences ranging from white (Taxon 1) to brownish gray (Taxon 2). Taxon 1 produced a hyalorhinocladiella-like asexual morph with simple and highly branched conidiophores, which often aggregate in loosely synnemata that were arranged either singly or in groups topped with white mucilaginous spore drops. Taxon 2 produced short mononematous conidiophores with allantoid conidia, and stalked club-shaped cells. A sexual morph could be induced in all isolates of Taxon 2; the most distinct features observed in both the herbarium specimens and the studied isolates were the short ascomatal necks and falcate ascospores with gelatinous sheaths. Sexual morph was not observed for Taxon 1 in any of the crosses done between different isolates. Morphological differences among these new taxa and the most closely related species are listed in Tables 3, 4, and discussed in the Notes under the new species descriptions in the Taxonomy section.

The optimal growth temperatures were 25 °C for Taxon 1 and 30 °C for Taxon 2. No growth was observed at 5 °C for Taxon 2.

### Phylogenetic analyses

Alignments for the *Ceratocystiopsis* data set of ITS1-5.8S-ITS2-28S contained 1278 characters and for the TUB2 512 characters (including gaps). Alignments for the ITS2-28S and the concatenated combined *Leptographium* data set of ITS1-5.8S-ITS2-28S+ACT+TUB2, TEF1-α, contained 637, and 13276 characters (including gaps), respectively. The exon/intron arrangement of the TUB2*Ceratocystiopsis* species complex data included exons 3, 4, 5 and 6, interrupted with introns 3, 4, and 5. The exon/intron arrangement of the TUB2*Leptographium* data included exons 3, 4, and 5/6, interrupted with introns 3 and 4, but lacking intron 5. The aligned TEF1-α gene region consisted of introns 3, 5 and exons 4/5, 6, while lacking intron 4. The alignment of the ACT dataset contained exons 5 and 6, interrupted with intron 5.

The ITS1-5.8S-ITS2-28S (Fig. 1) and ITS2-28S (Fig. 3) trees show the placement of the Polish isolates (referred to as Taxon 1 and Taxon 2) within the Ophiostomatales. Taxon 1 resided among sequences representing species that are members of *Ceratocystiopsis* (Fig. 1), while Taxon 2 grouped with other species in the *Leptographiumsensu lato* (Fig. 3). Taxon 1 was closely related with *C. pallidobrunnea* (Fig. 1), while Taxon 2 formed a separate lineage within *Leptographiumsensu lato* that included *Leptographium piriforme* and *Grosmannia crassivaginata* (Fig. 3). Taxon 1 had unique ITS1-5.8S-ITS2-28S sequences compared with other *Ceratocystiopsis* species (Fig. 1), while isolates of Taxon 2 had ITS2-28S sequences that were almost identical with sequences noted in *G. crassivaginata* (Fig. 3).

The MP, ML and BI analyses of the individual dataset (ITS2-28S, ACT, TUB2, TEF1-α) provided trees with similar topologies (data not shown). In the TUB2 tree (Fig. 2), Taxon 1 formed a well-supported lineage that clearly separated this newly proposed species from all the other known species in *Ceratocystiopsis* and the most closely related species *C. pallidobrunnea* (Fig. 2). The combined analyses of the ITS1-5.8S-ITS2-28S+TUB2+ACT+TEF1-α data grouped isolates of Taxon 2 in a lineage together with *L. piriforme* and *G. crassivaginata*, in agreement with the ITS2-28S tree. However, this taxon formed a well-supported lineage next to a clade containing *G. crassivaginata* (Fig. 4).

The six isolates of Taxon 1 obtained in this study were distinguished from *C. pallidobrunnea* using SNP analyses for each of the ITS1-5.8S-ITS2-28S, TUB2, TEF1-α gene region sequences. The total number of SNP differences between the six isolates and *C. pallidobrunnea* for all three genes was 166 (26 for ITS1-5.8S-ITS2-28S, 60 for TUB2, and 80 for TEF1-α). Little intraspecific sequence variation was found within 6 isolates of Taxon 1. Intraspecific variability of the ITS1-5.8S-ITS2-28S, TUB2 and TEF1-α genes was detected for Taxon 1 in one position, i.e. 387, two positions, i.e. 212, 217, and one position i.e. 482, respectively (Suppl. material 1: Tables S1, S2).

The 11 isolates of Taxon 2 obtained in this study were distinguished from *G. crassivaginata* using SNP analyses for each of the ITS1-5.8S-ITS2-28S, TUB2, TEF1-α, ACT gene region sequences. The total number of SNP differences between the 11 isolates and *G. crassivaginata* for all four genes was 59 (8 for ITS1-5.8S-ITS2-28S, 16 for TUB2, 25 for TEF1-α, and 10 for ACT). The intraspecific sequence variation was greater for 11 isolates of Taxon 2 than for Taxon 1. Intraspecific variability of the TUB2, TEF1-α, and ACT genes was detected for Taxon 2 in eight positions, i.e. 36, 82, 83, 87, 215, 230–232; nine positions, i.e. 14, 21, 31, 46, 101, 196, 272, 352, 549; and five positions, i.e. 402, 749, 754, 755, 766, respectively (Suppl. material 1: Table S3).

These results indicate that the six isolates of Taxon 1 within *Ceratocystiopsis* and the 11 isolates of Taxon 2 within *Leptographium* represent novel species.

## Taxonomy

The morphological characterization and phylogenetic comparisons based on six genetic loci, showed that two taxa associated with *D. alni* from Poland are distinct from each other and from other known taxa in the *Ophiostomatales*. Therefore, they are described here as new species:

### Taxon 1

#### Ceratocystiopsis synnemata

Taxon classificationFungiOphiostomatalesOphiostomataceae

B. Strzałka, R. Jankowiak & G. Hausner, sp. nov. MycoBank No: 835151

A43814EB-BC56-5E08-8F3D-E8DA28069ADB

Fig. 5

##### Etymology.

The epithet (synnemata) refers to the synnematous conidiomata formed by this fungus.

##### Type.

Poland, Paprocice, from *Dryocoetes alni* beetle infesting *Populus tremula*, 5 Oct 2018, *K. Miśkiewicz* (TUR 207995 http://mus.utu.fi/TFU.207995***holotype***, ex-holotype cultures: NRIF 16918DA = KFL 16918DA).

##### Description.

***Sexual morph***: not observed. ***Asexual morph***: hyalorhinocladiella-like. ***Conidiophores*** micronematous or macronematous. The micronematous conidiophores, hyaline, consist of conidiogenous cells arising singly from the vegetative hyphae (6–)8.6–16.4(–23.2) × (0.6–)0.9–1.3(–1.6) µm. The macronematous conidiophores are much larger, (14.5–)17.3–39.8(–76.9) µm long than the preceding forms and from a basal cell, (3.1–)5.3–11.2(–17) × (0.9–)1.1–1.9(–2.6) µm. The basal cells branch lateral or penicillate and form 1–5 branches (mostly 1–2) producing conidiogenous cells at their apices. ***Conidiophores*** often aggregate in loosely synnemata, (43.2–)52.3–86.4(–114.7) µm long, (2.4–)3.6–8.2(–12.9) µm wide at the tip. ***Conidia*** hyaline, smooth, unicellular, oblong-elliptical, (2.4–)2.8–3.5(–4) × (1–)1.1–1.3(–1.4) µm. ***Cultural characteristics***: Colonies with optimal growth at 25 °C on 2% MEA with radial growth rate 1.4 (± 0.1) mm/d, growth very well at 30 °C (1.3 mm/d) and 35 °C (1.0 mm/d). Colonies yellowish gray, margin smooth. Hyphae pale gray in color, smooth, submerged in the medium and aerial mycelium rare, not constricted at the septa, 0.4–2.6 (mean 1.1±0.6) µm diam., asexual morph moderately abundant, very abundant after adding twigs.

##### Host trees.

*Alnus incana*, *Populus tremula*

##### Insect vector.

*Dryocoetes alni*

##### Distribution.

Poland

##### Note.

*Ceratocystiopsis synnemata* can be distinguished from *C. pallidobrunnea* by the shape and size of the conidia. *Ceratocystiopsis synnemata* has shorter and oblong-elliptical conidia in contrast to the allantoid conidia of *C. pallidobrunnea* (*C. synnemata*: 2.4–4 ×1–1.4 µm; *C. pallidobrunnea*: 2.5–5 × 0.7–1.2 µm (Olchowecki and Reid 1974), 2–7 × 0.7–2.5 µm (Upadhyay 1981) (Table 3). In addition, *C. synnemata* produces conidiophores that aggregate into loosely arranged synnemata.

**Figure 5.**
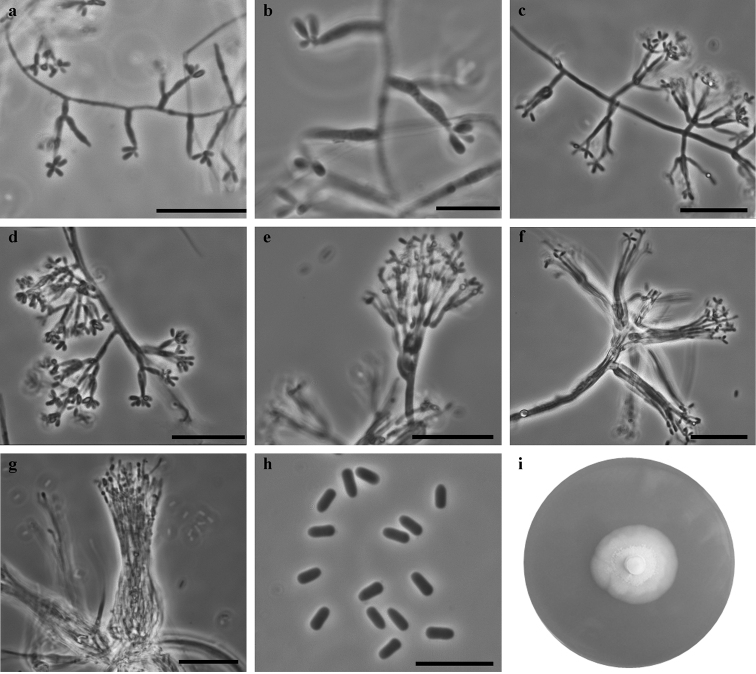
*Ceratocystiopsis synnemata* sp. nov. (NRIF 16918DA=KFL 16918DA) **a, b** micronematous conidiophores **c–e** macronematous conidiophores **f, g** conidiophores aggregate in synnemata**h** conidia **i** fourteen-day-old culture on MEA. Scale bars: 25 μm (**a**), 10 μm (**b**), 25 μm (**c–g**), 10 μm (**h**).

**Table 3.** Morphological comparisons of closely related species to *Ceratocystiopsis synnemata* sp. nov.

Species	*Ceratocystiopsis pallidobrunnea* (Olchowecki and Reid 1974)	*Ceratocystiopsis pallidobrunnea* (Upadhyay 1981)	*Ceratocystiopsis synnemata* sp. nov.
Sexual state	Present	Present	unknown
Ascomata base	40–60	40–75	
Ascomatal neck length (μm)	15–60	21.2–66	
Ascospore shape	allantoid or falcate with truncate ends in side view, cylindrical or fusiform with truncate ends in face view	falcate with truncate or obtuse ends in side view, fusiform or ellipsoid-fusiform in face view	
Ascospore size (in face view, μm)	(-3.5)4.5–7.5 × 0.7–1 excluding sheath	14–17.5(-22.5) × 1–1.5(-1.8) including sheath	
			
Conidial shape	allantoid or oblong with obtuse ends	cylindrical, allantoid	oblong-elliptical
Conidial size (μm)	2.5–5 × 0.7–1.2	2–7 × 0.7–2.5	2.4–4 × 1–1.4
Branched conidiophores	present, to 50 μm long	present	present, 76.9 μm long
Conidiophores aggregate into synnemata	absent	absent	present
Optimal growth temp on MEA	–	–	
Growth rate at optimum	–	–	
Host	*Populus tremuloides*	*Populus tremuloides*	*Alnus incana*, *Populus tremula*
Arthropods	unknown	unknown	*Dryocoetes alni*,
Distribution	Manitoba, Canada	Manitoba, Canada	Poland

### Taxon 2

#### Leptographium alneum

Taxon classificationFungiOphiostomatalesOphiostomataceae

B. Strzałka, R. Jankowiak & P. Bilański sp. nov.

6368BAB3-6455-5B38-9484-9C4BB9D50187

MycoBank No: 835146

Fig. 6

##### Etymology.

The epithet (alneum) refers to the species name of the bark beetle vector of this fungus, *Dryocoetes alni*.

##### Type.

Poland, Paprocice, from *Dryocoetes alni* beetle infesting *Populus tremula*, 2 Nov 2017, *K. Miśkiewicz*, (TUR 207557 http://mus.utu.fi/TFU.207557***holotype***, ex-holotype cultures: CBS 144091 = CMW 51789).

**Figure 6.**
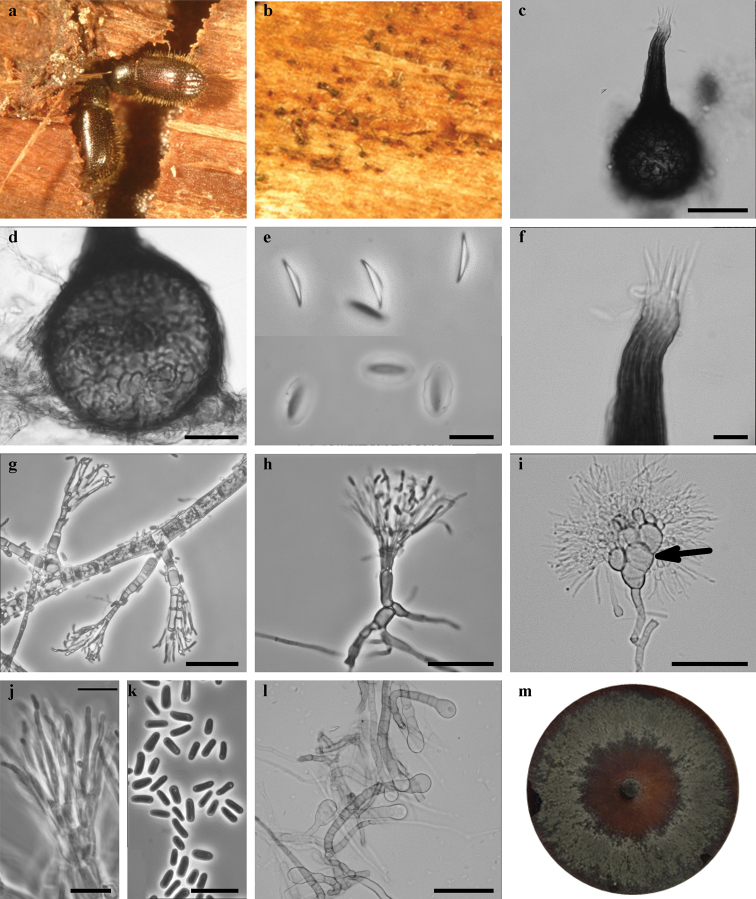
*Leptographium alneum* sp. nov. (CBS 144901) **a***Dryocoetes alni*-infested *Populus tremula* tree **b** galleries of *D. alni* with ascomata **c** ascoma **d** ascomatal base **e** ascospores **f** ostiolar hyphae **g–i** conidiophores, black arrow indicates barrel-shaped cells **j** conidiogenous **k** conidia **l** club-shape cells **m** fourteen-day-old culture on MEA. Scale bars: 50 μm (**c**), 25 μm (**d**), 10 μm (**e**), 10 μm (**f**), 25 μm (**g**), 25 μm (**h**), 50 μm (**i**), 10 μm (**j**), 10 μm (**k**), 50 μm (**l**).

##### Description.

***Sexual morph***: Ascomata developing after 30 d on sterilized *Populus* twigs when two mating types were paired: superficially or partly embedded in the agar or wood, single. Bases light brown to dark brown, globose, unornamented, (59–)66–90(–108) μm in diameter, necks dark brown, cylindrical, straight or curved, (58–)68–88(–114) μm long (excluding ostiolar hyphae), (18.7–)20.7–27.9(–31) μm wide at base, (10.2–)11.8–15.3(–17.8) μm wide at the tip. ***Ostiolar hyphae*** present, pale brown, straight, non-septate or sporadically one-septate, numerous, divergent, pointed at the tip, (14.6–)15.9–19(–22.7) μm long, 5 to 12 in number. ***Asci*** not seen. ***Ascospores*** one-celled, hyaline, falcate in side view, (7.4–)8.1–11.1(–14.3) × (1.2–)1.5–2.1(–2.4) μm; fusiform in face view, (6.9–)7.4–8.8(–10.3) × (1.8–)2–2.8(–3.3) μm; end view not seen, excluding hyaline gelatinous sheath, (8.9–)10–11.5(–12.2) × (4.5–)5.5–6.7(–7) μm in face view including sheath, accumulated in orange yellow-colored mass at the tip of the neck. Gelatinous sheath 0.5–3 μm thick, oval in face view.

***Asexual morph*: *conidiophores*** macronematous, arising directly from hyphae, single solitary, without rhizoidal hyphae at the bases, often with barrel-shaped or globose cells, (48.1–)59.3–84.2(–102.9) μm in length. ***Stipes*** erect, light olivaceous, 1–4 septate (mostly 2), (7.6–)14.3–39.2(–48.5) μm long (from first basal septum to below primary branches), (2–)2.4–5.4(–15.6) μm wide below primary branches, apical cell often strongly swollen, (3.2–)3.8–5.2(–6.1) μm wide at base, basal cell rarely swollen. Conidiophores often composed of barrel or globose cells. ***Conidiogenous apparatus*** (20–)26.5–38.6(–48.7) μm long (excluding conidial mass) consisting of 2–3 series of branches-type B (more than two branches) (Jacobs and Wingfield 2001). Primary branches light olivaceous, cylindrical or swollen, smooth, (5.1–)5.8–12.7(–23.1) × (1.2–)1.6–4.2(–6.8) μm. ***Conidiogenous cells*** hyaline, tapering from base to apex, (11.6–)13.2–19(–23.7) × (0.8–)0.9–2(–3.5) μm. ***Conidia*** hyaline, mostly allantoid, sometimes oblong to obovoid (3.2–)3.7–5.9(–9.7) × (0.8–)1–1.8(–2.8) μm, accumulating around the conidiogenous apparatus as a creamy mucilaginous mass.

***Cultural characteristics***: Colonies with optimal growth at 30 °C on 2% MEA with radial growth rate 8.8 (± 0.9) mm/d, good growth observed at 35 °C (8.3 mm/d) and better than at 25 °C (7.9 mm/d). Colonies brownish gray with distinct silvery gloss, margin smooth. Hyphae olive yellow in color, smooth, submerged in the medium and aerial mycelium abundant, not constricted at the septa, 1.1–5.5 (mean 2.5±1) µm diam., asexual morph very abundant, which gives a shade of gray. Club-shaped cells terminal on septate hyphal branches present, (11.5–)14.8–25.6(–33.3) × (7.7–)11.3–15.1(–18.2) μm, born on a multicelled stalk, (7.2–)14.7–82.4(–124.2) μm long, (4.4–)5.1–7.7(–9.7) μm wide below primary septa, (2.9–)4–6(–7.4) μm wide at base. Perithecia and asexual morph co-occur in culture.

##### Host trees.

*Alnus incana*, *Malus sylvestris*, *Populus tremula*

##### Insect vector.

*Dryocoetes alni*, *Scolytus mali*

##### Distribution.

Poland

##### Note.

Morphologically, *Leptographium alneum* differs from *Grosmannia crassivaginata* in having longer ascomatal necks (*L. alneum*: 58–114 µm: *G. crassivaginata*: 40–60 µm), and the presence of larger club-shaped cells (*L. alneum*: 11.5–33.3 × 7.7–18.2 µm; *G. crassivaginata*: 12–20 × 8–12 µm (Griffin 1968), 6.5–18.5 × 5–13.5 µm (CBS 119144), (Table 4). In addition, *L. alneum* has aseptate or sporadically 1-septate ostiolar hypahe, which are septate in *G. crassivaginata*. *Leptographium alneum* frequently produces conidiophores with barrel-shaped or globose cells, while in *G. crassivaginata* the cells of the conidiophore are slightly swollen at most. In contrast to *G. crassivaginata* (CBS 119144), *L. alneum* has larger conidia, especially in regard to width (*L. alneum*: (3.2–)3.7–5.9(–9.7) × (0.8–)1–1.8(–2.8) µm: *G. crassivaginata*: (2.4–)3.2–5(–8.1) × (0.7–)0.9–1.3(–1.7) µm) (Table 4). *Leptographium alneum* has brownish gray colony with silvery gloss cultures in contrast to the olive brown colored colonies of *G. crassivaginata* (isolate CBS 119144). The optimal growth on MEA for *L. alneum* and *G. crassivaginata* (isolate CBS 119144) is 30 °C. However, *L. alneum* grows much faster than *G. crassivaginata* (*L. alneum* 8.8 mm/d, *G. crassivaginata* 6.9 mm/d) and grows faster at 35 °C (8.3 mm/d) than at 25 °C (7.9 mm/d). In contrast, *G. crassivaginata* grows much faster at 25 °C (5.6 mm/d) than 35 °C (4.4 mm/d).

**Table 4.** Morphological comparisons of closely related species to *Leptographium alneum* sp. nov.

Species*	*G. crassivaginata* (Griffin 1968), holotype DAOM 110144)	*G. crassivaginata* (Jacobs and Wingfield 2001), holotype DAOM 110144)	*G. crassivaginata* (Upadhyay 1981), RWD 858, WIN(M) 69-12	*G. crassivaginata* (this study, CBS119144)	*L. piriforme* (Greif et al. 2006)	*L. alneum* sp. nov.
Sexual state	Present	Present	Present	Absent	Unknown	Present
Ascomata base	40–90	40–90	35–110			59–108
Ascomatal neck length (μm)	40–60	40–60	37–70 including ostiolar hyphae		–	58–114 excluding ostiolar hyphae
Ostiolar hyphae length (μm)	10–25, septate	–	septate			14.6–22.7, non-septate
Ascospore shape	Falcate in side view, fusiform in face view	Fusiform,	Falcate in side view, fusiform in face view		–	Falcate in side view, fusiform in face view
Ascospore size (in face view, μm)	5–7 × 1.excluding sheath, 10–11.5×5–6.5 including sheath	10–11 × 5–6 including sheath	9–12 × 5–7 including sheath		–	6.9–10.3 × 1.8–3.3 excluding sheath, 8.9–12.2 × 4.5–7 including sheath
Conidiophores length (μm)	to 50	25–105 (-120)	to 85	(28.6–)33.2–63.2(–109.1)		(48.1–)59.3–84.2(–102.9)
Stipe length (μm)		8–60(-85)		(5.6–)3.7–26(–58.6)	7–45.6	(7.6–)14.3–39.2(–48.5)
Conidiogenous apparatus length (μm)		15–55 (-60)		(22.3–)27–41.3(–54.7)		(20–)26.5–38.6(–48.7)
Conidial shape	Cylindrical to allantoid	Oblong to obovoid	Clavate, curved	Oblong to allantoids, often clavate	Curved	Cylindrical to allantoid
Conidial size (μm)	3–6 × 1–1.5	4–10×1–2	2.5–12 × 1–2	(2.4–)3.2–5(–8.1) × (0.7–)0.9–1.3(–1.7)	2.4–4.6 × 1.0–1.4	(3.2–)3.7–5.9(–9.7) × (0.8–)1–1.8(–2.8)
Club-shaped cells size (μm)	12–20 × 8–12 on short hyphal branches	–	9–23 × 7–14 on immersed hyphal branches	(6.5–)8.5–14.1(–18.5) × (5–)6.5–10.8(–13.5), born terminally or laterally on a non-septate or 2–3 septate stalk, 4.8–41.5 long, 2.5–7.3 wide below primary septa	14.4–31.2 × 7.2–16., borne on a one- to four celled stalk, 7.2–45.6 × 4.8–7.2	(11.5–)14.8–25.6(–33.3) × (7.7–)11.3–15.1(–18.2), born terminally on a multicelled stalk, 7.2–124.2 long , 4.4–9.7 wide below primary septa
Colony color and optimal growth temp on MEA	Brown, -	Olivaceous, 30,	Pale to dark brown or chaetura brown, -	Olive brown, 30	Light brown, 35	Brownish grey, 30
Radial growth rate (mm/d) at optimum	–	–		6.9 mm	–	8.8 mm
Host	*Picea mariana*, *Populus grandidentata*, *P. tremuloides*	*Picea mariana*, *P. glauca*, *Pinus resinosa*, *P. sylvestris*, *P. strobus*, *Fraxinus nigra*, *Populus grandidentata*, *P. tremuloides*	*Populus tremuloides*	unknown	Unknown (Greif et al. 2006); *Pinus sylvestris* (Jankowiak and Kolařík 2010); *Betula verrucosa* (Jankowiak et al. 2019c); *Populus tremula* (this study)	*Populus tremula*, *Malus sylvestris* (Jankowiak et al. 2019a, this study)
Arthropods	Unknown		–	Unknown	Coleoptera, Diptera, Araneae, Acari, Hemiptera, Lepidoptera, Collembola, Psocoptera, Trichoptera, and Hymenoptera: Formicidae, *D. alni* (this study)	*Dryocoetes alni*, *Scolytus mali*
Distribution	Ontario, Canada	Ontario, Canada,USA	Fort Collins, Colarado (USA), Monitoba (Canada)	Unknown	Alberta, Canada, Poland	Poland

*format ‘min-max’ or (min–)(mean-SD)–(mean+SD)(–max) for some morphological structures of *G. crassivaginata* (CBS119144) and *L. alneum* sp. nov.

## Discussion

This study identified two new species of ophiostomatoid fungi associated with *Dryocoetes alni* on *Alnus incana* and *Populus tremula* in hardwood ecosystems in Poland. DNA sequence comparisons and morphological features supported these as novel. The species were named *Ceratocystiopsis synnemata* and *Leptographium alneum*. The results confirm earlier findings that many species of the Ophiostomatales are associated with hardwood-infesting bark beetles in Poland (Jankowiak et al. 2017, 2018, 2019a; Aas et al. 2018).

The results of this study revealed one new species of *Ceratocystiopsis* bringing the total number of species in the genus to 17. The newly described species is morphologically similar to other species of *Ceratocystiopsis*, with hyalorhinocladiella-like asexual morph (De Beer et al. 2013a). In contrast to other *Ceratocystiopsis* species, *C. synnemata* produces simple as well as highly branched conidiophores reminiscent of *C. pallidobrunnea* (Olchowecki and Reid 1974; Upadhyay 1981) or *C. rollhanseniana* (Hausner et al. 2003). In addition, *C. synnemata* forms synnemata with loosely packed conidiophores that appear to be a unique feature of *Ceratocystiopsis*.

Ascomata in *Ceratocystiopsis* tended to be globose with short necks, and falcate ascospores surrounded by a gelatinous sheath (De Beer and Wingfield 2013). Generally, *Ceratocystiopsis* species produce perithecia in varying degrees of abundance and maturity (Upadhyay 1981; Plattner et al. 2009). *Ceratocystiopsis synnemata* did not form a sexual state in crosses between different isolates. That would suggest that this species is heterothallic or produces perithecia very sparsely.

Most of the formally described species of *Ceratocystiopsis* are known only from Pinaceae including those in the genera *Picea*, *Pinus* and *Pseudotsuga*. For example, in Poland, two species of *Ceratocystiopsis* have previously been reported: *C. minuta* and species of uncertain status, *C. alba*, which both have been isolated from spruce-infesting bark beetles (Jankowiak et al. 2009). Only *C. pallidobrunnea* was collected from hardwood tree species, *Populus tremuloides* in Canada (Olchowecki and Reid 1974; Plattner et al. 2009). The discovery of *C. synnemata* on *Populus tremula* in this study clearly show that *Ceratocystiopsis* species are also distributed in hardwood forest ecosystems in Europe.

A new species of *Leptographium* was discovered from *Dryocoetes alni* in this study. This new taxon is closely related to *Leptographium piriforme* and *Grosmannia crassivaginata* forming a well-supported lineage distinct from other species of *Leptographiumsensu lato.* All these three species have the curved conidia formed on short conidiophores, club-shaped cells, short-necked perithecia, and falcate, sheathed ascospores. These features clearly distinguish them from the other species recognized in the various species complexes currently recognized within *Leptographiumsensu lato*.

Based on DNA sequence comparisons, *L. alneum* described in this study is closely related to *G. crassivaginata*, a species described from *Picea mariana*, *Populus grandidentata* and *P. tremuloides* in Canada (Griffin 1968). Morphologically, *L. alneum* most closely resembles *G. crassivaginata*. The asexual morph of *L. alneum* produced short conidiophores, and stalked club-shaped cells, similar to those of *G. crassivaginata*. Other similarities are the presence of short-necked perithecia, and fusiform-falcate, sheathed ascospores. *Leptographium alneum*, however, differs from *G. crassivaginata*, in having longer ascomatal neck and larger club-shaped cells. Moreover, *L. alneum* has larger conidia than *G. crassivaginata* (CBS 119144), especially with regard to the width of the conidia. Other differences are the presence of barrel-shaped or globose cells that make up the conidiophores, which in *G. crassivaginata* are occasionally only slightly swollen. There are also differences in characteristics of cultures. *Leptographium alneum* has brownish gray cultures in contrast to olive brown culture of *G. crassivaginata* (isolate CBS 119144). Both species belong to fast-growing species on MEA, however *L. alneum* grows much faster than *G. crassivaginata*, especially at 35 °C.

*Leptographium* species are generally considered as saprotrophs or pathogens of conifer trees (Jacobs and Wingfield 2001). However, the results of the present study confirm previous Polish investigations that some of the *Leptographium* species have a close affinity to hardwoods in Europe. Recently, *L. betulae*, *L. tardum*, and *L. trypodendri* were collected from hardwood-infesting bark beetles in Poland (Jankowiak et al. 2017, 2018), while three other *Leptographium* species have been isolated and formally described from hardwood wounds (Jankowiak et al. 2018, 2019c).

There was no information on *D. alni*-associated fungi before 2019. Recent Polish research reported that only *L. alneum* (named as *Leptographium* sp. 7) is an associate of *D. alni* (Jankowiak et al. 2019a). However, the additional isolations conducted in 2018 demonstrated that this beetle species apart from *L. alneum*, was also associated with other ophiostomatoid species including *C. synnemata* and *L. piriforme*. Among them, only *C. synnemata* and *L. alneum* were commonly found in association with *D. alni*. The common occurrence of these species suggests their important role as fungal associates of *D. alni* in Poland. The results of the present study and other Polish findings (Jankowiak et al. 2019a) indicated that *C. synnemata* and *L. alneum* have been found only occasionally from other beetle species and therefore can be considered as regular and possible specific associates of *D. alni*. *Leptographium piriforme* is less specific and can be found with other beetle species (Jankowiak and Kolařík 2010) or hardwood wounds (Jankowiak et al. 2019c) in low numbers.

This work represents the most detailed survey of Ophiostomatales associated with *D. alni* in Europe. Two new species were described. Ophiostomatoid fungi on hardwoods have been relatively well investigated in Poland (Jankowiak et al. 2017, 2018, 2019a, b, c; Aas et al. 2018), but in other parts of Europe they are still poorly studied. In addition, many ophiostomatoid species have not been formally described and our study has contributed to filling this knowledge gap. The findings of this study clearly showed that the diversity and taxonomic placement of many members of the Ophiostomatales associated with hardwoods-infesting bark beetles in Europe are still poorly understood.

## Supplementary Material

XML Treatment for Ceratocystiopsis synnemata

XML Treatment for Leptographium alneum

## Supplementary materials

Supplementary material 1 Tables S1–S3 Data type molecular data Explanation note **Table S1.** Comparison of polymorphic sites of 18S–ITS1–5.8S–ITS2–28S and TUB2 genes of *Ceratocystiopsis pallidobrunnea* and Taxon 1. **Table S2.** Comparison of polymorphic sites of TEF1-α gene of *Ceratocystiopsis pallidobrunnea* and Taxon 1. **Table S3.** Comparison of polymorphic sites of 18S–ITS1–5.8S–ITS2–28S and protein-coding genes of *Grosmannia crassivaginata* and Taxon 2. https://binary.pensoft.net/file/422418This dataset is made available under the Open Database License (http://opendatacommons.org/licenses/odbl/1.0/). The Open Database License (ODbL) is a license agreement intended to allow users to freely share, modify, and use this Dataset while maintaining this same freedom for others, provided that the original source and author(s) are credited.Beata Strzałka, Robert Jankowiak, Piotr Bilański, Nikita Patel, Georg Hausner, Riikka Linnakoski, Halvor Solheim
