# Supplementary material for: Two new species of Ophiostomatales (Sordariomycetes) associated with the bark beetle Dryocoetes alni from Poland
Source: MycoKeys. 2020 Jun 17;68:23–48. doi: 10.3897/mycokeys.68.50035 (PMC7314864; doi:10.3897/mycokeys.68.50035)
Supplement: Supplementary material 1 — Tables S1–S3 [file mycokeys-68-023-s001.docx]

**Table S1** Comparison of polymorphic sites of 18S–ITS1–5.8S–ITS2–28S and TUB2 genes of *Ceratocystiopsis pallidobrunnea* and Taxon 1. Numbers written vertically above columns indicate the relative positions in the alignments

| Species | Strains | Genes* | |
| --- | --- | --- | --- |
|  |  | 18S-28S | TUB2 |
|  |  | $$$$$$$$$$$$###########!!!! | eeeiiiiiiiiiiiiiiiiiiiiiiiiiiiiiiiiiiiieeeeeiiiiiiiiieeeeeeeeee |
|  |  | 1111  111111111334455555550111  333017777799784445556678011  568944567801878920490504757 | 1111111111111112222233333344  1133344444456666667777777777888990001122223777890111235589933  192834512346704567890123456789035272390423484078441247915884778 |
| Taxon 1 | KFL16216DA | **TACCAA----CCGTCGCACGTG-TCCC** | **ACTCCTCTTGTCG-----------------CTAACCTGCAACCTCCACTCGTTTCCCTTTAC** |
|  | KFL13418DA | **TACCAA----CCGTCGCACGTG-TCCC** | **ACTCCTCTTGTCG-----------------CTAACCTGCAACCTCCACTCGCTCCCCTTTAC** |
|  | KFL149a18DA | **TACCAA----CCGCCGCACGTG-TCCC** | **ACTCCTCTTGTCG-----------------CTAACCTGCAACCTCCACTCGTTTCCCTTTAC** |
|  | KFL149b18DA | **TACCAA----CCGCCGCACGTG-TCCC** | **ACTCCTCTTGTCG-----------------CTAACCTGCAACCTCCACTCGTTTCCCTTTAC** |
|  | KFL16918DA | **TACCAA----CCGCCGCACGTG-TCCC** | **ACTCCTCTTGTCG-----------------CTAACCTGCAACCTCCACTCGTTTCCCTTTAC** |
|  | KFL17718DA | **TACCAA----CCGCCGCACGTG-TCCC** | **ACTCCTCTTGTCG-----------------CTAACCTGCAACCTCCACTCGTTTCCCTTTAC** |
| *C. pallidobrunnea* | WIN(M) 51 | **AC-TGCAAACAAACACAGACCATCATT** | **GTCTTCTGCTCT-CGTAGCTTTCTACGAATTCTGGTCCGGCATCTTCTCAACCCTTTCCCCT** |

*different signs indicate: $ – ITS1, # – ITS2, ! – 28S, i – introns, e – exons.

**Table S2** Comparison of polymorphic sites of TEF1-α gene of *Ceratocystiopsis pallidobrunnea* and Taxon 1. Numbers written vertically above columns indicate the relative positions in the alignments

| Species | Strains | Gene* |
| --- | --- | --- |
|  |  | TEF1-α |
|  |  | iiiiiiiiiiiiiiiiiiiiiiiiiiiiiiiiiiiiiiiiiiiiiiiiiieeeeeeeiiiiiiiiiiiiiiiiiiiiiiii |
|  |  | 11111111112222222222222222222222222223333344444444444444444444444444  122344555556811124445580122222223333444444555556782345711344455566666667777888889  835901234690367911675627714567890123136789012346488165912723424512345782489123494 |
| Taxon 1 | KFL16216DA | **TT-CTCCTCGTAACAGTTTTGTTTCA----------TT---------GTATCATCGACGCCCCTACAGGCTTCGTCCCTCT** |
|  | KFL13418DA | **TT-CTCCTCGTAACAGTTTTGTTTCA----------TT---------GTATCATCGACGCCCCTACAGGCTTCGTCTCTCT** |
|  | KFL149a18DA | **TT-CTCCTCGTAACAGTTTTGTTTCA----------TT---------GTATCATCGACGCCCCTACAGGCTTCGTCCCTCT** |
|  | KFL149b18DA | **TT-CTCCTCGTAACAGTTTTGTTTCA----------TT---------GTATCATCGACGCCCCTACAGGCTTCGTCCCTCT** |
|  | KFL16918DA | **TT-CTCCTCGTAACAGTTTTGTTTCA----------TT---------GTATCATCGACGCCCCTACAGGCTTCGTCTCTCT** |
|  | KFL17718DA | **TT-CTCCTCGTAACAGTTTTGTTTCA----------TT---------GTATCATCGACGCCCCTACAGGCTTCGTCCCTCT** |
| *C. pallidobrunnea* | WIN(M) 51 | **CCT---TCTAATGTCAACC-ACCGGCACATCACCACGCTCATCTTTTTCTCAGCTAGTTTAT--TTGCATCCT--GTTGTG** |

*different signs indicate: i – introns, e – exons.

**Table S3** Comparison of polymorphic sites of 18S–ITS1–5.8S–ITS2–28S and protein-coding genes of *Grosmannia crassivaginata* and Taxon 2. Numbers written vertically above columns indicate the relative positions in the alignments

| Species | Strains | Genes* | | | |
| --- | --- | --- | --- | --- | --- |
|  |  | 18S-28S | TUB2 | TEF1-α | ACT |
|  |  | $$$$$$#! | iiiiiiiiiiiiiiiiii | iiiiiiiiiiiiiiiiiiiiiiiiiiiiiiie | eeeeeeiiiiiiii |
|  |  | 1  11111140  22233339  57823436 | 1222222  34567788880122333  65960123474559012 | 1111111112222223333357  12333345670000666691234790134548  41134769031234248962097290051294 | 2345677777777  13304344556788  51326689457601 |
| Taxon 2 | CBS144905 | **ACTTCTTC** | **TTATAA---ATTTT---** | **-CTAGCCAT-----GC--GTTTGGTTAATTGT** | **GCGGCCCCT-GATG** |
| Taxon 2 | CBS144904 | **ACTTCTTC** | **CTATAAAA-GTTTT---** | **CCTAGC-AT-----GC--GTTTGGTTAATTGT** | **GCGGCCCCCCGATG** |
| Taxon 2 | CBS144903 | **ACTTCTTC** | **TTATAA---ATTTT---** | **-CTAGCCAT-----GC--GTTTGGTTAATTGT** | **GCGGCCCCCCGATG** |
| Taxon 2 | CMW52070 | **ACTTCTTC** | **TTATAA---ATCTTTTC** | **CTTAGTCAT-T---GC--GTTTGGTTAATTAT** | **GCGGCCCCCCGATG** |
| Taxon 2 | CBS144902 | **ACTTCTTC** | **TTATAA---ATTTT---** | **CCTAGCCAT-----GC--ATTTGATTAATCAT** | **GCGCCCCTC-GTTG** |
| Taxon 2 | CMW52069 | **ACTTCTTC** | **TTATAAA--ATTTT---** | **CCTAGCCAT-----GC--ATTTGATTAATCAT** | **GCGGCCCCC-GTTG** |
| Taxon 2 | KFL8617RJDA | **ACTTCTTC** | **TTATAA---ATCTTTTC** | **CCTAGCCAT-----GC--ATTTGATTAATCAT** | **GCGGCCCCC-GTTG** |
| Taxon 2 | CBS144901 | **ACTTCTTC** | **TTATAAA--ATTTT---** | **CCCAGCCAT-----GC--ATTTGATTAATCAT** | **GCGGCCCCCCGATG** |
| Taxon 2 | KFL9117RJDA | **ACTTCTTC** | **TTATAAA--ATTTT---** | **CCCAGCCAT-----GC--ATTTGATTAATCAT** | **GCGGCCCCCCGATG** |
| Taxon 2 | KFL88616RJSM | **ACTTCTTC** | **TTATAA---ATTTT---** | **-CTAGCCAT-----GC--ATTTGATTAATCAT** | **GCGCCCCTC-GTTG** |
| Taxon 2 | CBS144900 | **ACTTCTTC** | **TTATAA---ATTTT---** | **CCTAGC-AT-----GC--ATTTGATTAATCAT** | **GCGGCCCCC-GATG** |
| *G. crassivaginata* | CBS119144 | **CTCCTCCT** | **TAGCCCAAAACTCCT-C** | **CCTCATC--CTTTTCTATTCACAA--TGCCAC** | **AGCGGTTC--AAAA** |

*different signs indicate: $ – ITS1, # – ITS2, ! – 28S, i – introns, e – exons.
